# Gaps in knowledge and practices of malaria prevention in Francophone African immigrants in Metropolitan Edmonton

**DOI:** 10.1186/s12936-022-04210-w

**Published:** 2022-06-21

**Authors:** Taylor A. Hanna, Ali Ahmed, Rémi Vincent, Kongnon Sangué Coulibaly, Youssef Ahmed, Ryland Petrick, Etienne Vincent, Mélanie El Hafid, Michel T. Hawkes, Srilata Ravi, Sedami Gnidehou

**Affiliations:** 1 Faculty Saint-Jean, University of Alberta, Edmonton, Canada; 2 Faculty of Science, University of Alberta, Edmonton, AB Canada; 3 Department of Pediatrics, Faculty of Medicine, University of Alberta, Edmonton, AB Canada; 4 Department of Medical Microbiology and Immunology, Faculty of Medicine, University of Alberta, Edmonton, AB Canada

**Keywords:** Imported malaria, Knowledge, Prevention, Immigrant, Non-endemic settings

## Abstract

**Background:**

Important knowledge gaps exist in the understanding of the management of the risks of imported malaria in Canada among Francophone immigrants from sub-Saharan Africa (FISSA). The aim of this cross-sectional study was to investigate the malaria related-knowledge, attitude and practices (KAP) of FISSA in Edmonton, where these immigrants are in an official minority language situation and the impact of language barriers on these factors.

**Methods:**

A structured survey was used to examine the KAP of 382 FISSA in the Edmonton area from 2018 to 2019. Fisher’s Exact Test was applied to determine if there were associations between knowledge of malaria and different risk factors.

**Results:**

Almost all FISSA (97%) had an accurate knowledge of fever as the key symptom of malaria. Interestingly, 60% of participants identified bed nets as a preventive method and only 19% of participants had accurate knowledge of malaria transmission. An accurate knowledge of symptoms was significantly associated with a high perceived risk of contracting malaria [odds ratio (OR) 4.33, 95% confidence interval (CI) 1.07–20.62]. Furthermore, even though 70% of FISSA had a high perceived risk of contracting malaria in endemic regions, only 52% of travellers had a pre-travel medical encounter. Importantly, language was not the predominant reason for not seeking pre-travel medical advice, although 84% of respondents chose French as their official language of preference when seeking medical advice. Having a French-speaking physician was correlated with satisfactory prevention knowledge (OR 1.96, 95% CI 1.16–3.35). With respect to health-seeking behaviour, 88% of respondents with a child < 5 years of age would seek medical care for fever in the child after travel to sub-Saharan Africa (SSA).

**Conclusion:**

This study highlights that factors other than knowledge, risk assessment, and language might determine the lack of compliance with pre-travel medical encounters. It underscores the need for effective strategies to improve this adherence in minority settings.

**Supplementary Information:**

The online version contains supplementary material available at 10.1186/s12936-022-04210-w.

## Background

Malaria is a life-threatening disease caused by parasites that are transmitted to humans through the bites of infected female *Anopheles* mosquitoes [1]. The World Health Organization (WHO) estimates that in 2020 there were 241 million malaria cases worldwide, and 627,000 deaths [1]. Approximately ninety-five percent of malaria cases and deaths were reported in the region of SSA where *Plasmodium falciparum*, the species responsible for the most lethal form of the disease, is prevalent [1].

Imported malaria (IM) or travel-associated malaria is promoted by population movements and is a growing public health challenge in many industrialized non-endemic countries [2]. Even though the disease is preventable, every year 30,000 IM cases are reported in non-endemic regions globally [3–5]. In Canada, the annual number of cases is approximately 420 [5, 6]. Travellers returning from SSA make up a disproportionate share of these infections, ranging between 70–79% [7–9] and this is consistent with the global burden of malaria. Moreover, similar to European countries and the United States, Canada’s reported IM cases are densely concentrated within regions exhibiting a steady growth of immigrants [8, 10, 11].

Strong correlations between risk of contracting malaria and other factors such as length, type of travel and travel behaviour have been established [12, 13]. Despite progress, several lines of evidence have highlighted immigrants visiting friends and relatives (VFR) in their country of origin, particularly in SSA, as being at greatest risk of acquiring IM infections [11, 14]. Not only does this population tend to stay in rural areas for extended periods of time when visiting friends and relatives, they are also less likely to take appropriate pre-travel measures; these include seeking medical advice or taking prophylaxis before travelling to endemic areas, including but not limited to SSA [9]. One explanation is that based on immigrants’ former life experiences in SSA, interventions like prophylaxis are unfamiliar concepts. Secondly, similar to most immigrants from malaria-endemic regions, SSA immigrants often overestimate their immunity to malaria [9] or may simply prefer taking preventive measures upon arrival. Unfortunately, counterfeit anti-malarial drugs are commonly found over the counter in SSA, and may pose even more health risks [15–17].

A study has shown that Alberta has the third highest incidence rate of severe malaria infections within Canada [8]. Severe malaria is defined by the clinical features established by the WHO including impaired consciousness or coma, respiratory distress and severe anaemia.

Canadian National Health Care (CNHC) system is government sponsored with its services under each province’s regulation (Reviewed in [18, 19]). All populations including citizens, permanent residents and immigrants regardless of income, employment or health have the accesses to health services without cost, portability between provinces and low-cost prescription drugs. Nevertheless, a high proportion of the population (three-quarter) has a private supplemental health insurance, either through an employer or a secondary insurer (Reviewed in [19]. Unfortunately, several healthcare services including travel-related medication and vaccines are expensive and not covered by most basic health plans [20], making access to care a major barrier to pre-travel care and malaria prophylaxis for VFR.

Furthermore, impacts of language barriers on accessibility, safety and quality of healthcare services are well documented [21, 22]. Canada is a multicultural country where both English and French are official languages. Nevertheless, Canadians living in official language minority settings, including Francophone living outside the French speaking province of Quebec, could face similar language related-barriers to health care services as other language minorities [23].

Health disparities among immigrant populations are associated with limited English proficiency and discordant languages between providers and immigrant patients [24–26]. For populations whose preferred official language is French, a limited access to health-related services in French was reported [23]. However, it is worthwhile noting that the number of immigrants, especially French-Speaking (francophone) immigrants from SSA (FISSA) who are considered at particular risk of IM, has significantly increased in Alberta in the last decade [27]. Therefore, FISSA who are living in predominantly English-speaking communities represent a unique population of interest when studying preventive knowledge and practice of IM in minority settings. Challenges related to language barriers in minority settings when trying to access medical advice before a trip to malaria endemic settings may increase this population’s susceptibility to IM infections.

There are no studies on the prevention and management of IM by FISSA in Canada outside of Quebec. Therefore, knowledge gaps exist in the understanding of the management of malaria in FISSA in linguistic minority settings such as the metropolitan region of Edmonton (capital of Alberta). The percentage of FISSA using pre-travel medical counselling is not known and the knowledge, attitudes and practices (KAP) of IM prevention are rarely studied in this specific group in Alberta.

This study is the first to characterize FISSA malaria-related knowledge and symptoms. The precautions that these populations took prior to travelling to endemic areas and the methods (hospital consultation, self-medication, peer counseling) they would use post-travel in case of fever were also examined.

## Methods

### Study design

A cross-sectional survey of FISSA who live in the Edmonton Metropolitan Region was conducted to characterize malaria-related knowledge, attitudes, and preventive practices prior, during and after travel to endemic areas. The primary survey outcome of interest was health-seeking behaviour after travel. Specifically, this study measures the proportion of FISSA respondents with a child < 5 who would seek care for the child in case of fever arising within a month of return from travel to sub-Saharan Africa. Secondary survey outcomes of interest were related to knowledge of malaria symptoms, transmission and other parameters associated to preventive attitude and practice.

### Study setting, participants and inclusion criteria

Edmonton (53°34′N, 113°31′W), capital of Alberta, Canada is a non-endemic region for malaria. Nevertheless, Alberta is the province with the 3rd highest incidence rate of severe malaria cases within Canada [8]. The total population of the Edmonton Metropolitan area was 1.387 million during the period of the study (2018–2019) [26] with a growing francophone immigrant population in the last decade. From 2000 to 2019, populations of FISSA have risen by 1650% in the Edmonton Metropolitan Region.

The study included francophone adults (≥ 18 years old; male and female) who are first or second-generation immigrants from SSA countries and who lived in the Edmonton region at the time of study. In this study, Francophone was defined as a person who speaks French as their mother tongue [28]. Participants were excluded if they had never heard of malaria or indicated that they were not of sub-Saharan African origin.

VFR travellers were defined as first- or second-generation immigrants who are ethnically different or racially distinct from the majority population of their current country of residence and who return to their country of origin to visit friends and relatives [9, 29].

### Sampling

#### Estimation of sample size

For estimation of sample size, the focus was on self-reported health care-seeking practices (intention) with respect to the respondent or a child aged 5 years or younger with febrile illness returning from travel to sub-Saharan Africa. A standard sample size calculation was assessed as previously described [30]. The sample size calculation indicated that 384 participants would be needed to estimate with ± 5% precision the proportion of respondents who would go or bring a febrile child to the doctor in case of fever after returning from a malaria-endemic area, at the α = 0.05 level of significance.

#### Sampling technique

Participants were recruited between September 2018 and March 2019 from the University of Alberta’s francophone faculty Campus Saint-Jean (CSJ), an immigrant settlement agency called the Centre d'Accueil et d’Établissement du Nord de l’Alberta (CAE), and the office of Dr. Denis Vincent, all of which are located in Edmonton’s French Quarter. Posters related to the project were displayed at CSJ, CAE and the office of Dr. Denis Vincent. Copies of paper-based questionnaires were also made available in those sites. Four counselors at CAE presented the project at the targeted population visiting the centre. When needed, participants were referred to the last author for any questions. At CSJ, three research assistants were hired to approach the participants. Dr. Vincent’s secretary proposed the study to patients visiting their clinic and who may be interested in the study. In addition, information sessions were also held at the CAE during social events by the research team. These sessions helped to explain the project to the targeted demographic and allowed for interested participants to ask any questions and participate in the study. Participants were also recruited directly within the community by word of mouth. Some participants either completed the paper version in person and handed it or completed the paper-based questionnaire at home and brought it to the last author’s office. Other participants completed the survey electronically through the internet either in person or remotely.

### Survey questionnaire

A secondary level 94-item questionnaire written in French due to our target demographic being francophones was composed of 70 questions and 22 sub-questions with a variety of questions in each section reflective of the area of interest. The choice of questionnaire items was guided by a need for contextually appropriate questions for French-speaking immigrants in Canada. The questions were elaborated using surveys from previous studies [31–33]. Lead authors with tacit knowledge of the circumstances, culture and language of the FISSA, chose the appropriate wording of the questions and adapted the content of the questionnaire to the conditions of the FISSA in Edmonton.

The survey was pre-tested on 5 participants from the target group to evaluate the understanding of questions and to identify any issue. Consequently, the questionnaire was adjusted based on outcomes of the pre-testing. Data from the pre-testing were not included in the analysis. The survey was made available through a QR (Quick Response) code on posters located throughout the Campus Saint-Jean campus, an immigrant settlement agency known as the CAE, and a doctor’s office that is known to have FISSA patients. The QR code is a system designed to embed digital links in documents and is a useful way to allow access to online data via smartphones. In this study, the QR code from the posters linked to an electronic version of the survey on the KoboToolbox software. Of the 409 participants, 63% completed the paper-based survey in person and 37% through the internet by using the QR code.

### List of variables

The questionnaire had 5 sections covering participants’ socio-demographic information, detailed travel history (not limited to SSA) and corresponding pre-travel, on-travel and post-travel prevention measures for malaria (e.g., pre-travel consultations, malaria prophylaxis, and use of bed nets) and cultural influences. Questions on malaria’s causes, transmission, symptoms and prevention were also asked. The variable includes:

1. Demographics: The socio-demographic section contained 19 questions and 2 sub-questions. It included factors such as city of residence, age, gender, country of birth, education level, language, having health insurance and having a family physician.
2. Travel: The travel section included 7 questions with 7 sub-questions related to trips that respondents made. They were asked to list travels taken to malaria-endemic regions, such as sub-Saharan Africa, South East Asia, or Latin America, within the last 5 years, and to give the reasons for travelling. Subsequent questions were based on a child at or under the age of 5 accompanying the respondents which probed the medical outcomes depending on whether or not the child developed symptoms and tested positive for malaria upon their return to Canada.
3. Knowledge of malaria: The knowledge section included 11 questions with 1 sub-question on the causes, symptoms and mode of transmission of malaria. Participants were asked to choose from a list of possible sources they used for information regarding malaria (multiple selections possible). Questions regarding malaria symptoms, transmission, and prevention were used to analyze the respondents’ understanding of the disease. Using a list of symptoms, including 1 detractor symptom (itchiness), participants were asked to agree whether malaria was associated with each symptom (“yes” or “no”) (multiple selections possible). Participants were considered to have sufficient knowledge of symptoms if they chose “yes” to fever, as fever is the defining symptom of malaria. The list of possible methods of transmission was in the same structure as the list of symptoms with multiple selections possible. Participants were considered to have adequate knowledge of methods of transmission if they simultaneously recognized “mosquito bite” and “*Plasmodium falciparum*” as methods of transmission and rejected “drinking dirty water” and “dirty environment”. Methods of prevention were collected using an open-ended question. Comprehensive knowledge of malaria was defined as possessing adequate knowledge of symptoms, transmission, and prevention.
4. Attitudes and practices: The attitudes and practices section included 8 questions with 7 sub-questions related to health, malaria prophylaxis while travelling and other preventive measures. A range of attitudes were analysed including the severity of the disease as well as the level of reliance on the Canadian healthcare system in the form of pre-travel consults and post-travel care in the event of presentation of malaria symptoms. Using open-ended questions, participants were asked to state the reason for not seeking pre-travel medical advice or not using precautions upon arrival in endemic regions. Participants were given scenario questions to analyse their post-travel practices when presenting with symptoms of malaria or generally feeling unwell after a trip to endemic regions.
5. Culture and Society: The last section, which contained 25 questions and 7 sub-questions focused on culture and society. Participants were asked about their beliefs regarding malaria and how that impacted their medical encounter with the Canadian health care system. Data from this section wasn’t included in this manuscript.

Remarkably, of the 409 participants only 7 participants failed to answer all questions of the survey.

### Data sources and processing

Participants filled out the survey either electronically (using the KoboToolbox application) or using a paper version. Data from all paper submissions were manually entered into the KoboToolbox database and a second team member verified the input to check for accuracy, consistency and missed values. Answers from both entries were compared and discrepancies were corrected. A Microsoft Excel spreadsheet containing data from all 409 participants was generated from a KoboToolbox (Version 2.019.52) database. Twenty-seven entries were excluded for the following reasons: 5 participants answered the survey both manually and electronically and discrepancies between answers were found, 10 entries featured unclear or unreadable answers, and 7 participants failed to complete the questionnaire. Thus, data from 382 respondents were considered for analysis (Additional file 1: Fig. 1S). Open ended questions were analysed by extracting key elements from the answers that were relevant to the question. For instance, the only malaria preventive methods considered were bed nets, anti-malarial medication, and anti-mosquito spray.

### Estimation of comprehensive knowledge of malaria

The participants’ comprehensive malaria knowledge was assessed using a modified scoring system that was described elsewhere [34, 35]. Comprehensive knowledge of malaria was assessed by combining data from the following categories: malaria symptoms, transmission and prevention. Participants were considered as having accurate knowledge of malaria symptoms if they identified fever as the main symptom of malaria. Accurate knowledge of malaria transmission meant that participants identified “*Plasmodium falciparum”* and “mosquito bites” as causes of malaria, while simultaneously rejecting two incorrect means of transmission “drinking dirty water” and “dirty environment”. Similar identification and rejection were described in previous studies [6]. Lastly, those who identified bed nets as a preventive method were considered as having accurate knowledge of malaria prevention. Questions regarding selected criteria including knowledge of malaria symptoms, transmission and prevention methods were scored and pulled together. For each respondent, the mean score was computed to determine the overall comprehensive knowledge of malaria. Participants scored average and above were considered as having a comprehensive knowledge of malaria.

### Statistical analysis

Variables analysed included sociodemographic factors such as age, gender, education, health insurance and language, along with factors like reason for travel, country visited, travel with children aged 5 years or under, having a French-speaking physician, and comprehensive malaria knowledge, attitudes and practices. These factors were considered given their relevance to the objectives of the study. Statistical analysis was conducted using R software (version 3.6.3, R Core Team 2020). For descriptive statistics, numbers (percentages) for proportions were used. Two-by-two contingency tables were used to test for associations between knowledge of malaria and FISSA characteristics such as having a family physician and having health insurance. Associations between knowledge of malaria and key attitudes related to malaria treatment and severity were also tested. Odd ratios (cross-product ratio of 2 by 2 table entries) and their confidence intervals (CI) were calculated using Fisher’s exact test. A 95% CI was used and the cut-off value for statistical significance was p ≤ 0.05.

## Results

Approximately 500 eligible FISSA were approached and 409 participated to the study (response rate 81.8%; Additional file 1: Fig. S1).

Participants were recruited in the city of Edmonton and in 4 of Edmonton metropolitan cities (401 in the city of Edmonton, 4 in Sherwood Park, 2 in Leduc, 1 in Spruce Grove and 1 in St-Albert). The general demographic, health and travel characteristics of the study population are shown in Table 1. Participants were predominantly first-generation immigrants from sub-Saharan Africa (96%). All recruits in this study referred to themselves as francophone. They were well-educated and especially trained in francophone schools and universities. The largest proportion (76%) of them had a post-secondary degree and 23% a high school diploma or less. These data suggest a relatively high fluency in French. Most (65%) of the respondents had a personal or family history with the disease. Even though 84% of respondents chose French as the language in which they are most at ease, 57% of them indicated that they did not have a French-speaking family physician. Of the 382 respondents, 155 (41%) travelled to malaria-endemic regions between 2013 and 2019. SSA was the most common travel destination, and trips had a mean duration of 34 days. In addition, 9% of travellers travelled to malaria-endemic countries in Asia, and 12% in South America (Table 1, Additional file 1: Fig. S2). Visiting friends and relatives was the most cited reason for travel (Table 1).

**Table 1:** General demographic, health and travels characteristics of the study population

Parameter	Frequency (n)	Percentage^(a)^ (%)
Gender		
Female Male Other	183 198 1	48 52 0
Age (33.8 ± 11.261)^b^		
18–29 30–44 45–59 Older than 60	171 142 60 9	45 37 16 2
Birth country		
Cameroun Ivory Coast Democratic Republic of Congo Other in Sub Saharan Africa^c^ Other/no answer^d^	83 71 50 161 17	22 19 13 42 4
Education		
Post-secondary learning High school diploma or less	291 88	76 23
Language^e^		
French English French and English Other	321 35 17 9	84 9 4 2
Contracted malaria prior to moving to Canada		
Yes No I don’t know	248 118 16	65 31 4
Have a family physician		
Yes No	295 87	77 23
Have a French speaking family physician^f^		
Yes No No answer	123 169 3	42 57 1
Number of respondents who have travelled to an endemic region in the last 5 years^g^	155	41
Average trip length (Days, mean ± SD) Africa (n=224, 33.8 ± 28.3) Other continents (n=27, 14.4 ± 28.3)		
Continents frequented by travellers^h^		
Africa Asia Latin America	144 9 12	93 6 8
Purpose of travel^h,I^		
Visiting friends and relatives Other	136 31	88 20

^a^Percentage indicates the proportion of respondents who fall into each category out of 382

^b^Mean ± SD

^c^Other countries of birth in Sub-Saharan Africa includes Benin n= 9 (6%), Burkina Faso n=10 (6%), Burundi n=18 (11%), Central African Republic n=3 (2%), Congo n=11 (7%), Gabon n=5 (3%), Ghana n=1 (1%), Guinea n=33 (20%), Mali n=7 (4%), Mauritania n=7 (4%), Niger n=5 (3%), Rwanda 8 (5%), Senegal n=34 (21%), Tchad n=2 (1%), and Togo n=8 (5%)

^d^16 participants were second generation immigrants who had at least one parent born in Sub Saharan Africa. Countries of birth of these participants included: Canada (7), France (6), Germany (2), and the United States (1). 1 participant did not respond

^e^Language in which respondents are most at ease

^f^Calculations are only for those (295) who have a family physician

^g^Endemic Regions include Sub Saharan Africa, Latin America, and South-West Asia

^h^n=155 participants who travelled to an endemic region

^i^Participants who indicated having travelled multiple times may have been included in more than one category.

While most FISSA had a personal or family history with the disease and 97% of them identified fever as a key symptom, accurate knowledge of malaria transmission and prevention were less common, with values of 19% and 60%, respectively. Only 14% of participants demonstrated comprehensive malaria knowledge, meeting the combined criteria for accurate knowledge of symptoms, transmission, and prevention (Table 2).

**Table 2:** Knowledge of malaria symptoms, transmission, and prevention

Parameter	Accurate Frequency, n = 382 (Percentage %)	Poor Frequency, n = 382 (Percentage %)
Knowledge of malaria symptoms		
Fever	370 (97)	12 (3)
Knowledge of malaria transmission		
Mosquito bite	74 (19)	308 (81)
* Plasmodium falciparum*	366 (96)	16 (4)
Reject drinking dirty water	164 (43)	218 (57)
Reject dirty environment	329 (86)	53 (14)
	225 (59)	157 (41)
Knowledge of malaria prevention		
Bed nets	229 (60)	152 (40)
Comprehensive knowledge of malaria^a^		
Knew key symptom (fever), means of transmission, and prevention measures	52 (14)	330 (86)

^a^Participants are considered to have comprehensive knowledge of malaria if they simultaneously:

-Mention fever as a symptom of malaria

-Mention “*Plasmodium falciparum*” and “mosquito bite” as a means of transmission

-Reject “drinking dirty water” and “dirty environment” as a means of transmission

-Mention “bed nets” as a preventive measure

Several majors’ factors can impact respondent’s malaria knowledge. In comparison with other respondents, respondents with a level of education higher than secondary level were significantly more likely to have an accurate comprehensive knowledge of malaria (OR 2.50, 95% CI 1.01–7.42) as well as an accurate knowledge of malaria symptom (OR 3.46, 95% CI 0.90–13.32), (Table 3). Furthermore, a prior exposure to malaria was significantly associated with knowledge of malaria prevention (OR 2.58, 95% CI 1.60–4.16) (Table 3).

**Table 3:** Risk factors associated with knowledge of malaria

Parameter	Symptoms		Transmission		Prevention		Comprehensive	
	OR (95% CI)	*P *value	OR (95% CI)	*P *value	OR (95% CI)	*P *value	OR (95% CI)	*P *value
Post secondary education	3.46 (0.9–13.32)	0.037*	1.89 (0.93–4.19)	0.089	1.45 (0.88–2.40)	0.14	2.50 (1.01–7.42)	0.048*
Had previously experienced malaria	3.05 (0.81–12.5)	0.062	1.24 (0.68–2.32)	0.57	2.58 (1.60–4.16)	5.19E-5*	1.70 (0.81–3.85)	0.18
Health insurance	3.04 (0.51–12.93)	0.12	4.89 (1.21–43.80)	0.02^*^	1.46 (0.71–3.01)	0.30	6.56 (1.05–271.39)	0.04*
Perceived risk of contracting malaria^b^	4.33 (1.07–20.62)	0.02^*^	1.55 (0.82–3.08)	0.19	1.29 (0.78–2.12)	0.33	1.55 (0.74–3.52)	0.24
Malaria as a mortal disease^b^	3.24 (0.85–15.02)	0.07	0.91 (0.52–1.62)	0.79	1.11 (0.69–1.79)	0.73	0.90 (0.74–1.75)	0.75
Family physician	1.13 (0.19–4.68)	0.74	1.20 (0.63–2.41)	0.64	1.76 (1.06–2.94)	0.02*	1.48 (0.67–3.60)	0.38
French speaking family physician^c^	0.35 (0.06–1.70)	0.17	0.54 (0.27–1.02)	0.05	1.96 (1.16–3.35)	0.01*	0.70 (0.33–1.43)	0.32
Part of family with children aged 5 and under	1.63 (0.34–15.6)	0.74	1.41 (0.76–2.56)	0.23	1.20 (0.73–2.02)	0.47	1.46 (0.72–2.87)	0.30
Salary^(d)^	5.93 (0.65–284)	0.10	2.16 (1.09–4.39)	0.02^*^	0.85 (0.49–1.50)	0.59	2.01 (0.94–4.50)	0.06

^a^Participants are considered to have comprehensive knowledge of malaria if they simultaneously: mention fever as a symptom of malaria, mention “*Plasmodium falciparum*” and “mosquito bite” as a means of transmission, reject “drinking dirty water” and “dirty environment” as a means of transmission, and mention “bed nets” as a preventive measure

^b^Participants were asked to assess the risk of malaria contraction and mortality on a scale of 1 to 10. High perceived risk corresponds to ratings >5

^c^Compares participants who have a French speaking family physician with those who have a non-French speaking family physician

^d^Compares participants who have a yearly salary/income of 50 000$ or more with those who have a yearly salary/income of less than 50 000$

^*^P value ≤ 0.05. Statistically significant

Malaria preventive medications at Travellers Health Services (THS) are expensive [31, 36]. Furthermore, an appointment for a family at THS can be quite costly especially with vaccines and drugs [37–39]. The lack of insurance coverage represents a major barrier for immigrants when looking for pre-travel medical counselling [31, 40]. Having health insurance was significantly associated with knowledge of malaria transmission (OR 4.89, 95% 1.21–43.80) and overall comprehensive knowledge (OR 6.56, 95% CI 1.05–271.39) (Table 3).

A study has pointed out that a failure to perceive malaria as a personal risk and lack of knowledge of preventive measures were important factors in non-protection [41]. A high perceived risk of contracting malaria was significantly associated with accurate knowledge of symptoms (OR 4.33, 95% CI 1.07–20.62). Similar to other major factors, professional healthcare providers play a pivotal role in malaria knowledge’s transmission and VFR preventive behaviours (Reviewed in [42]). Having a family physician was significantly associated with knowledge of malaria prevention (OR 1.76, 95% CI 1.06–2.94), (Table 3). Importantly, since the goal of this study was to characterize the KAP of FISSA living in an English-speaking environment, having a French family physician was associated with satisfactory knowledge about prevention (OR 1.96, 95% CI 1.16–3.35) (Table 3).

Overall, the FISSA presented satisfactory attitudes related to the severity, risk assessment, diagnosis and treatment of malaria (Additional file 1: Table S1). The majority of the participants had a high perceived risk of contracting malaria (70%) and risk of malaria as a mortal disease (61%). Despite having incorrectly answered some questions about whether in Canada, anti-malarial medication should be available at the pharmacy without the need for a doctor’s prescription and whether malaria can be recognized without using a blood test, most respondents correctly answered the questions determined to measure attitude about the severity and risk of assessment of the disease. Of the 382 respondents, approximately 77% agree/strongly agree that malaria can be severe in both children and adults. Moreover, most (66%) disagree/strongly disagree that malaria can be treated at home without the opinion of a physician.

Searching for medical advice before travelling to malaria endemic regions is one of the hallmarks of satisfactory pre-travel practices [43]. In Canada, Travel health medical advices can be provided directly by primary care providers, travel clinics, pharmacies and other healthcare providers ([44, 45]. Moreover, patients can go directly to THS without a referral (Reviewed in [42]). In this study, despite 70% of FISSA having a high perceived risk of contracting malaria, only 52% of travellers booked a pre-travel medical consultation (Table 4). Travellers who sought medical advice received that information from physicians (29%), travel clinics (18%), and pharmacies (5%) (Table 4). One third of travellers did not seek pre-travel medical advice (34%). Respondents’ most common reasons for not seeking healthcare advice were that it was not worth it (31%), they were not used to doing it (31%), or they were already informed (15%) (Table 4). Similar reasons were provided by respondents who indicated not taking any travel precautions before or during their stay in endemic areas (Table 4).

**Table 4:** Pre-travel and during travel practices among travellers

Parameter	Frequency, n=155 who travelled to endemic regions (Percentage %)
Pre-travel practices	
Type of advice sought	
Canadian doctors	45 (29)
Travelers health services	28 (18)
Pharmacy	7 (5)
Other^a^	23 (14)
No advice sought	52 (34)
Reason for not seeking healthcare practitioner’s advice before leaving Canada (n=52)	
Not worth it^b^	16 (31)
Not used to do it	16 (31)
Already informed	8 (15)
Language barrier^c^	1 (2)
Other	11 (21)
Preventive methods before leaving Canada	
Anti-malarial drugs from health professional in Canada^d^	73 (47)
Mosquito repellent cream and incense	53 (34)
Had put a bed net in my suitcase	26 (17)
Non-prescription drugs sold in Canada	9 (6)
Other	9 (6)
No pre-travel precautions taken	48 (31)
Practices during travel	
Effective preventive methods used when arrived in malaria endemic areas	
Bed nets	64 (41)
Anti-malarial medication^e^	24 (15)
Anti-malarial drugs from health professional in Canada	34 (22)
Anti-mosquito cream/spray	36 (23)
Other	18 (12)
No preventive measures taken	27 (17)
Reason for not using preventive methods in arrival country (n=27)	
Not worth it	10 (37)
Was not informed/Not used to doing it	8 (30)
Prior immunity	2 (7)
Other	7 (26)

All 382 participants answered to questions that are presented in this table. But for the relevance of the topic, only data from those who travelled to endemic areas were considered for analysis.

^a^Participants answered “yes” but listed encounters that were abroad (n=9), non-medical (n=5), said they did not know (n=7), or did not specify (n=1).

^b^Two participants who had seen a doctor also indicated that the appointment had not been worth it.

^c^One participant who had seen a doctor also indicated that there had been significant language barriers during the encounter.

^d^34/73 (47%) participants who were prescribed anti-malarial drugs from a health professional in Canada did use them during stay in malaria endemic areas, 39/73 (53%) participants did not.

^e^Of the 155 participants, 58/155 used anti-malarial medication, 34/58 (59%) were prescribed medication by a health professional in Canada. 24/58 (41%) participants received anti-malarial medication through other ways. In the table above, we display 24/155 (15%) for all those who used anti-malarial medication that were not prescribed from a health professional in Canada.

THS is most knowledgeable when it comes to knowledge related to prevention against IM [37, 46]. Interestingly, this study demonstrated that only 18% of travellers used THS. Those who had not sought medical advice had predominantly received advice or information from a physician in the endemic area, the internet, or from family and friends.

Another important finding from this study is the fact that of all 155 participants who traveled, 73 received an anti-malarial prescription from health providers in Canada. Forty-seven percent (34/73) of this sub population used them upon arrival. Twenty-four of the 155 participants also used anti-malarial medication at arrival obtained through other ways. Overall, only 37% (58/155) of participants who travelled used anti-malarial medication during their stay in malaria endemic areas (Table 4). The reasons why those who were prescribed the drugs did not use them need to be addressed in further studies.

Statistically significant association were observed between FISSA who had pre-travel consultations and the use of preventive methods (e.g. packing beds nets, anti-malarial medication and/or repellent) both before (p < 0.001) and during (p < 0.01) travel to malaria endemic regions (Table 5). In addition, a strong association was found between FISSA who had a family physician and those who sought pretravel advices (OR 4.23, 95% CI 1.56–12.87). However, no significant associations were found between accurate knowledge of malaria symptoms, transmission, prevention, comprehensive knowledge, high perceived risk of malaria as a potentially lethal disease, high perceived risk of contracting malaria, prior exposure to malaria, having a French speaking family physician, level of education and having had a pre-travel consultation or using preventive measures upon arrival (Table 5). Moreover, 90% of travellers who had a medical consult and took preventive measures prior to their travel also used preventive methods upon arrival to their travel destination.

**Table 5:** Factors associated with malaria preventive methods among travellers

Parameter	Pre-travel consultation with Canadian health professional		Used pre-travel preventive methods		Used preventive methods during stay in malaria endemic region	
	OR (95% CI)	*P *value	OR (95% CI)	*P *value	OR (95% CI)	*P *value
Pre-travel consultation with Canadian health professional	N/A	N/A	10.74 (4.29–30.04)	5.45E−9*	3.39 (1.28–9.78)	8.5E–3*
Knowledge of malaria symptoms	2.48 (0.13–149)	0.59	0.74 (0.01–9.48)	1	4.74 (0.33–68.23)	0.15
Knowledge of malaria transmission	0.85 (0.35–2.08)	0.68	1.12 (0.45–3.03)	1	3.82 (0.87–35.18)	0.07
Knowledge of malaria prevention	0.73 (0.35–1.5)	0.40	1.01 (0.47–2.14)	1	1.47 (0.58–3.70)	0.39
Comprehensive knowledge of malaria^(a)^	0.97 (0.36–2.72)	1	1.32 (0.45–4.39)	0.81	5.62 (0.83–241.75)	0.08
High perceived risk of contracting malaria^(b)^	0.69 (0.23–1.90)	0.49	0.85 (0.25–2.50)	0.81	1.78 (0.51–5.51)	0.37
High perceived risk of malaria as a mortal disease^(b)^	0.98 (0.44–2.19)	1	0.44 (0.85–15.02)	0.07	0.38 (0.09–1.24)	0.10
Had previously experienced malaria	0.87 (0.35–2.10)	0.84	0.92 (0.35–2.25)	1	1.56 (0.53–4.26)	0.32
Post secondary education	0.78 (0.26–2.25)	0.64	0.66 (0.18–2.04)	0.61	0.20 (0.005–1.35)	0.13
Family physician	4.23 (1.56–12.87)	0.002*	1.57 (0.60–3.96)	0.37	1.68 (0.53–4.79)	0.29
French speaking family physician^(c)^	0.93 (0.41–2.13)	1	0.81 (0.35–1.92)	0.69	0.90 (0.32–2.66)	1

^a^Participants are considered to have comprehensive knowledge of malaria if they simultaneously: mention fever as a symptom of malaria, mention “*Plasmodium falciparum*” and “mosquito bite” as a means of transmission, reject “drinking dirty water” and “dirty environment” as a means of transmission, and mention “bed nets” as a preventive measure

^b^ Participants were asked to assess the risk of malaria contraction and mortality on a scale of 1 to 10. High perceived risk corresponds to ratings >5

^c^Compares participants who have a French speaking family physician with those who have a non-French speaking family physician

^*^P value ≤ 0.05. Statistically significant

Seeking hospital care for febrile children, relatives or oneself following a trip to an endemic region and informing the physician about a trip to an endemic area are considered as satisfactory practices against IM [47, 48]. In this study, we observed that these practices were common and positively shared by more than 50% and 88% of respondents, respectively (Table 6).

**Table 6:** Post-travel practices among travellers

Parameter	Frequency, n=155 who travelled to endemic regions (Percentage %)
If you or a member of your family does not feel well after a trip to an endemic region^a^, you:*	
Go to hospital emergency	73 (47)
Go to family doctor	64 (41)
Use a home treatment	25 (16)
Go to pharmacy	15 (10)
Consult traditional practitioner	3 (2)
Other	5 (3)
If you or your family member has a fever after a trip to an endemic region^a^ you:*	
Go to hospital emergency	77 (50)
Go to family doctor	62 (40)
Use a home treatment	20 (13)
Go to pharmacy	21 (14)
Consult traditional practitioner	5 (3)
Other	6 (4)
After a trip to an endemic region^a^ , you or your child do not feel well and you go to the hospital, you:	
Tell the doctor about your trip	136 (88)
Do not tell the doctor about your trip	4 (3)
You do not know	14 (9)
No answer	1 (1)
After a trip to an endemic region^a^ , you or your child do not feel well and you go to the hospital. The doctor:	
Asks you about your trip to an endemic region	100 (65)
Does not ask you about your trip to an endemic region	13 (8)
You do not know	41 (26)
No answer	1 (1)

All 382 participants answered to questions that are presented in this table. But for the relevance of the topic, only data from those who travelled to endemic areas were considered for analysis

^a^Endemic Regions include Sub Saharan Africa, Latin America, and South-West Asia

^*^Multiple answers could be given

## Discussion

This study presents the results of a cross-sectional study of FISSA in Edmonton to assess the malaria related-KAP in minority settings within Canada. One of the key findings of this study is that despite the fact that a significant number of FISSA had a level of education higher than secondary level, a high proportion of them had a poor comprehensive knowledge of malaria. Moreover, this unique population of VFR travellers was not prone to seek pre-travel medical advice. Remarkably, some travellers do not systematically use during their stay the anti-malarial drugs that were prescribed by Canadian health professional. In addition, a large proportion of travellers used anti-malarial drugs for treatment or prophylaxis that had either been prescribed or obtained probably from other sources in their travel destination. Although malaria KAP among FISSA in linguistic minority settings was scarcely reported in Canada, these data are in keeping with findings from studies that state poor adherence to pre-travel medical advice among VFR travellers from Canada [7, 9] and other non-endemic industrialized countries [31, 32]. Curiously, while having a French family physician was associated with knowledge of malaria prevention, such lack of adherence to pre-travel preventive measures was not influenced by a language barrier despite FISSA living in a French language minority setting.

While level of education was associated to the awareness of fever as malaria symptom and comprehensive knowledge, prior exposure to malaria was significantly associated to accurate knowledge of malaria prevention. The high awareness of fever as a malaria symptom might be due to previous self-experience of malaria since most respondents mentioned past personal or familial history of the disease. This hypothesis was consistent with findings reported by other studies [33, 49]. Nevertheless, having past personal or familial history of malaria was not associated with an accurate comprehensive knowledge of malaria. These data suggest that accurate comprehensive knowledge of malaria, as described in this study, does not necessarily rely on having previous personal or familial experiences of the disease.

A study has observed that having satisfactory knowledge of preventive malaria measures is not directly reflected in appropriate adherence to preventive malaria practices [49]. In this study, associations between FISSA’s level of knowledge and any preventive measure were not found. Furthermore, having a high perceived risk of contracting the disease during a stay in an endemic area was not sufficient to seek pre-travel medical advice. These data suggest that factors other than knowledge and high perceived risk assessment contribute to good anti-malaria preventive practices. The lack of adherence with a pre-travel medical encounter may be due to the FISSA having a little trust in the knowledge of tropical diseases of health professionals in Canada [50, 51]. Consistent with this hypothesis, seeking advice from a healthcare professional was cited as futile. Other contributing factors may relate to the expensive cost of anti-malarial drugs that are not covered by provincial health insurance plans [20, 31].

Although malaria recommendation is available in Canada, there can be ambiguity in anti-malarial preventive advice among health professionals. Canadian physicians, traveller’s health services and pharmacists were identified as providers of pre-travel malaria advice. Whether uniform and accurate malaria preventive advice was provided by all healthcare professionals is questionable since studies have identified limited malarial knowledge among physicians [48, 50] and pharmacists [44] in Canada. Yet, the differences that were observed in behaviours and adherence to preventive measures might also arise from the variation among pre-travel medical advices sources.

Studies reported associations between language barriers and lack of seeking health advice [25, 26]. While most FISSA indicated French as their official language of preference and only 42% of them had a French-speaking family physician, surprisingly language barriers were noted in only 2% of participants as the leading deterrent in seeking a healthcare practitioner’s advice before travelling. This suggests that FISSA probably rely on other resources for travel-related health information. This hypothesis is in line with our findings that showed that the internet, physicians in malaria endemic regions, and family and friends as other sources of malaria information for travelers who have not sought medical advice in Canada before travelling. Nevertheless, the importance of language in FISSA KAP related to malaria was not completely excluded as having a French speaking family physician was found to increase the odds of having appropriate knowledge of malaria prevention. Additional studies that involve FISSA and healthcare providers are needed to better characterize the impact of language among FISSA on malaria attitudes and practices in minority settings like Alberta.

Collectively, in terms of identifying a significant association between major factors that can potentially impact malaria KAP such as education, previous exposure to malaria, having a family physician, having a French speaking physician, and jointly knowledge of malaria and preventive practices, results from this study reveal the complexity of characterizing FISSA’s KAP in minority settings. As such, understanding how FISSA socio-demographic conditions and knowledge of malaria are translated into preventive measures is important to be considered in the control of IM.

Overall this study provides interesting insight into FISSA knowledge of malaria and prompts us to ask key questions on specific effective ways to (i) disseminate information about malaria preventive knowledge, attitude and practices to the FISSA community and to (ii) probe travelers who are reluctant for a pre-travel medical visit. Studies have recommended diverse strategies to tackle these questions in immigrants’ groups. These interventions included education initiatives to target high risk populations of travelers [52], training of travel health providers for a better communication with immigrants [52, 53], financial incentives such as cost-reduction strategies or efforts to limit out-of-pocket costs [53], improvement of patients communication and experience [53, 54], and changing negative perception about health providers expertise [53]. While these strategies are important, in order to create a culturally sensitive educational prevention program we propose to preferentially include a strong collaboration with ethnic media, community leaders, presidents of Francophone African associations, Francophone African health professionals as well as organizations that usually provide services (education, health, settlement etc.) to FISSA. Programmes that mobilize ethnical partners can play a critical role in the adoption of preventive behaviors against IM. These are in keeping with findings that revealed an effectiveness and increase of the use of health services through promotional health messages by mass media and ethnic community organizations [54]. Frequent educational workshops should be organized in collaboration with these organizations to train FISSA on health access in minority settings as well as preventive measures against infectious diseases that are at particular risk in FISSA group. In the case of IM, since prophylaxis is not generally affordable for all immigrants in Canada and has unpredictable quality in SSA, using systematically treated bed nets and window screens during a stay in malaria endemic settings should be prioritized and be part of the information as bed nets are cheaper and available in SSA. Importantly further studies asking FISSA to propose interventions that might better address the lack of using pre-travel measures are needed.

This study has certain limitations. Firstly, although a high proportion of FISSA had an accurate knowledge of malaria symptom and a high perceived risk of contracting malaria; the length, format, and reading level of the survey might have probably selected for a more educated, more knowledgeable about some aspects of malaria and lower barriers to accessing care subgroup of FISSAs. Moreover, while it is well known that not all immigrants necessarily return to their country of origin, travellers in this study may not represent the breadth of FISSA travellers. Additionally, while pre-travel malaria advice queries varied with regards to not only preventive measures but also for several information related to healthcare in endemic areas, findings from this study do not necessarily reflect the pre-travel malaria concerns of all FISSA. Finally, an ability to comment on FISSA preventive malaria knowledge, and on how a high adherence to pre-travel medical consultation can directly mitigate the incidence of IM into Canada is hindered by the lack of the estimation of travellers who acquired IM or who get a malaria related symptom after a trip in SSA regions.

## Conclusion

Although most of the respondents had a personal or family history with malaria and a satisfactory behaviour related to seeking hospital care for febrile children, relatives or oneself following a trip to an endemic region, there remain many gaps. The proportion of FISSA who had a satisfactory comprehensive knowledge of the disease as described in this study is low. Furthermore, this unique population of VFR travellers was not prone to seek pre-travel medical advice. Those who sought pre-travel medical do not systematically use during their stay the anti-malarial drugs that were prescribed by Canadian health professionals. Given what is known about impacts of language barriers on accessibility of healthcare services, curiously, in this study the lack of adherence to pre-travel preventive measures was not influenced by a language barrier despite FISSA living in a French language minority setting. Further studies that collect data from VFR travellers originally from different malaria epidemiological settings including SSA, Asia, Oceania and Latin America and living in minority linguistic settings in Canada are required to better study the impacts of language barrier in adherence to malaria preventive measures.

## Supplementary Information

**Additional file 1: Figure S1**. Flow chart of participants. **Figure S2.** The distribution of travel destination of FISSA who had traveled. **Table S1**. Participant’s attitudes regarding malaria.

## Data Availability

The datasets used and/or analysed during the current study are available from the corresponding author on reasonable request.

## Abbreviations

FISSA: Francophone immigrants from sub-Saharan Africa
KAP: Knowledge, attitude and practices
OR: Odd ratio
CI: Confidence interval
SSA: Sub-Saharan Africa
IM: Imported malaria
VFR: Visiting friends and relatives
CSJ: Campus Saint-Jean
CAE: Centre d'Accueil et d’Établissement du Nord de l’Alberta
QR: Quick response
THS: Travellers Health Services
