# Supplementary material for: Gaps in knowledge and practices of malaria prevention in Francophone African immigrants in Metropolitan Edmonton
Source: Malar J. 2022 Jun 21;21:197. doi: 10.1186/s12936-022-04210-w (PMC9215031; doi:10.1186/s12936-022-04210-w)
Supplement: Supplementary file 1 — Additional file 1: Figure S1. Flow chart of participants. Figure S2. The distribution of travel destination of FISSA who had traveled. Table S1. Participant’s attitudes regarding malaria. [file 12936_2022_4210_MOESM1_ESM.pptx]

## Slide 1
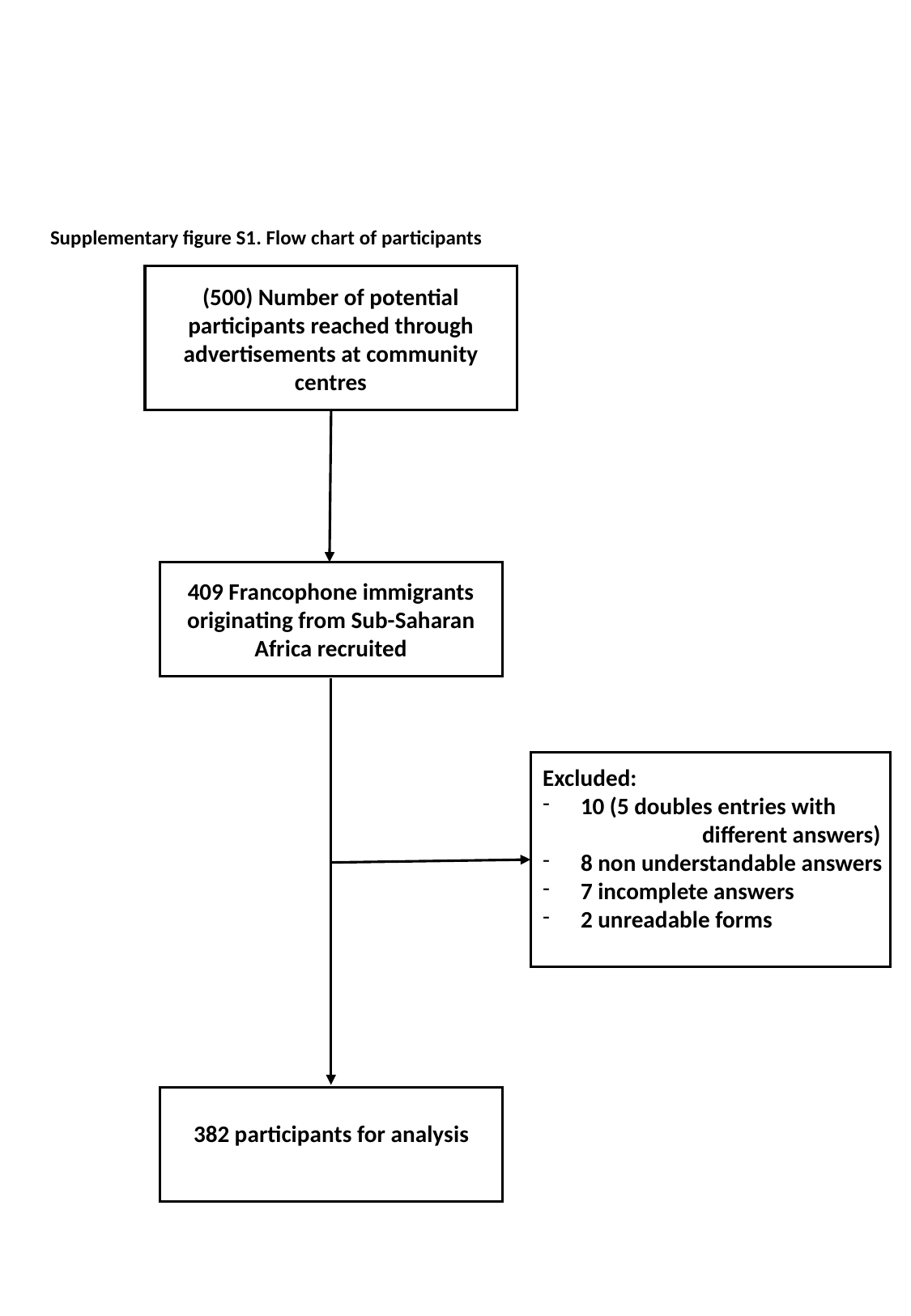

Supplementary figure S1. Flow chart of participants
(500) Number of potential participants reached through advertisements at community centres
409 Francophone immigrants originating from Sub-Saharan Africa recruited
Excluded:
10 (5 doubles entries with 	different answers)
8 non understandable answers
7 incomplete answers
2 unreadable forms
382 participants for analysis

## Slide 2
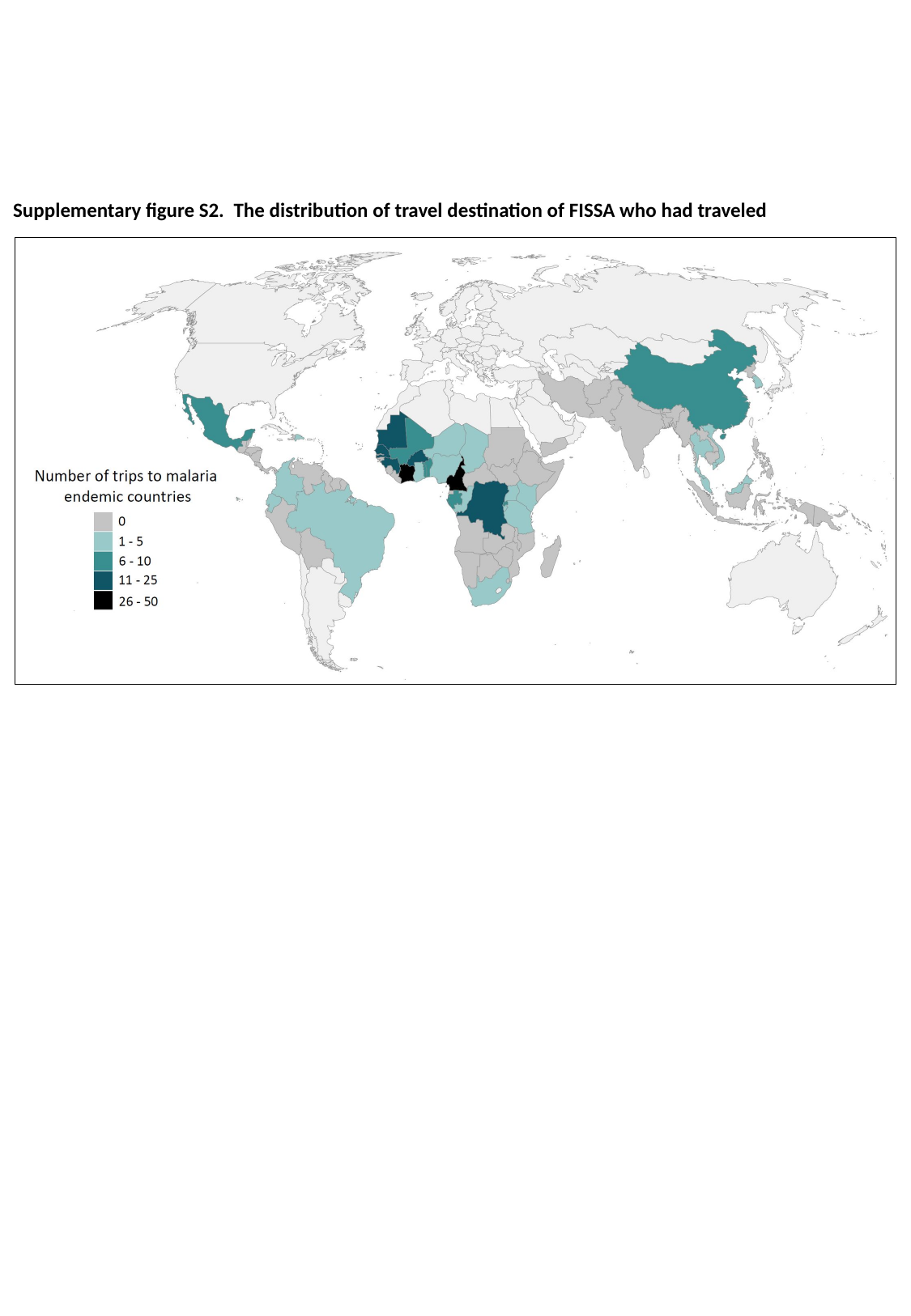

Supplementary figure S2. The distribution of travel destination of FISSA who had traveled

## Slide 3
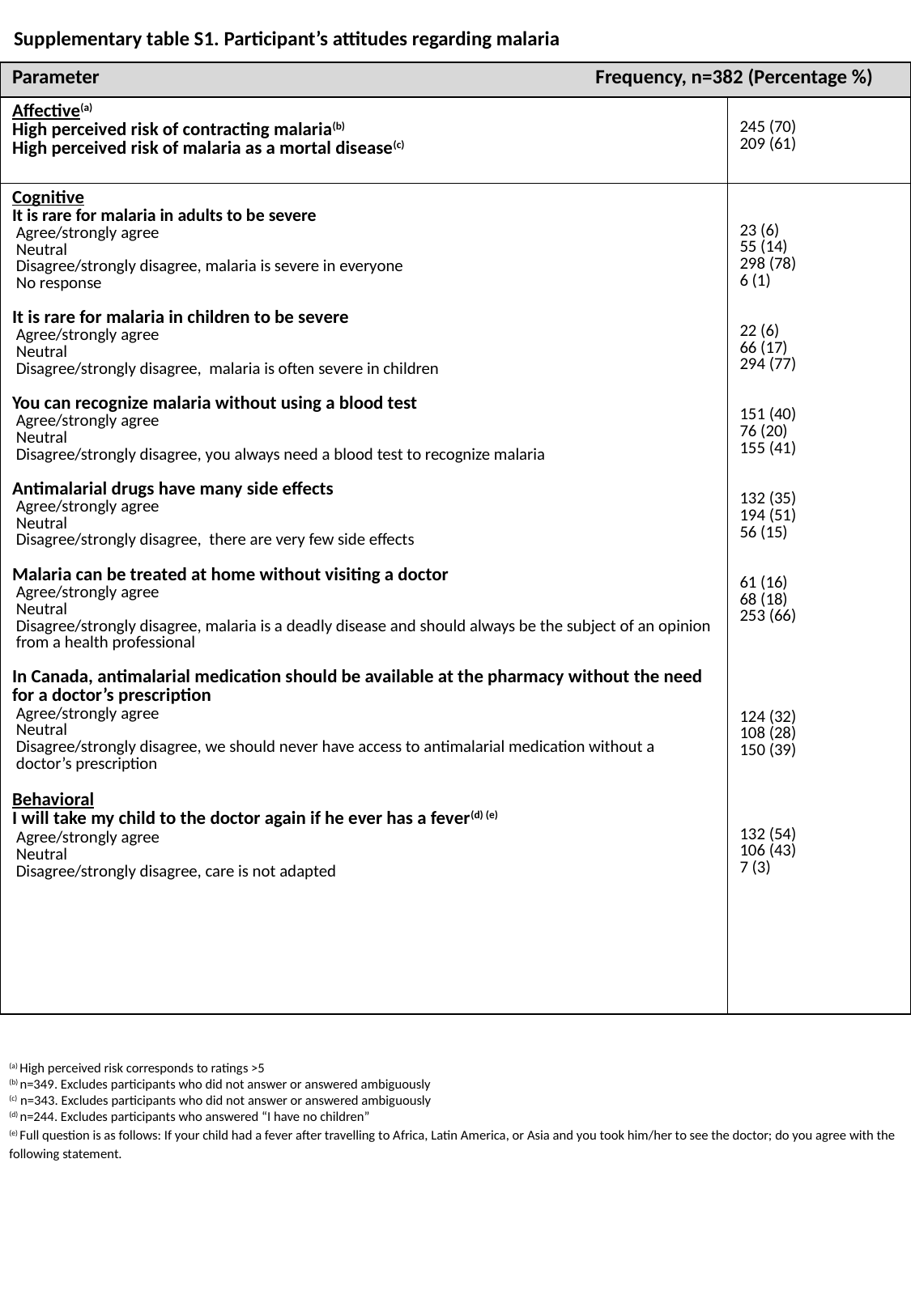

Supplementary table S1. Participant’s attitudes regarding malaria
| Parameter Frequency, n=382 (Percentage %) | |
| --- | --- |
| Affective(a) High perceived risk of contracting malaria(b) High perceived risk of malaria as a mortal disease(c) | 245 (70) 209 (61) |
| Cognitive It is rare for malaria in adults to be severe Agree/strongly agree Neutral Disagree/strongly disagree, malaria is severe in everyone No response It is rare for malaria in children to be severe Agree/strongly agree Neutral Disagree/strongly disagree, malaria is often severe in children You can recognize malaria without using a blood test Agree/strongly agree Neutral Disagree/strongly disagree, you always need a blood test to recognize malaria Antimalarial drugs have many side effects Agree/strongly agree Neutral Disagree/strongly disagree, there are very few side effects Malaria can be treated at home without visiting a doctor Agree/strongly agree Neutral Disagree/strongly disagree, malaria is a deadly disease and should always be the subject of an opinion from a health professional In Canada, antimalarial medication should be available at the pharmacy without the need for a doctor’s prescription Agree/strongly agree Neutral Disagree/strongly disagree, we should never have access to antimalarial medication without a doctor’s prescription Behavioral I will take my child to the doctor again if he ever has a fever(d) (e) Agree/strongly agree Neutral Disagree/strongly disagree, care is not adapted | 23 (6) 55 (14) 298 (78) 6 (1) 22 (6) 66 (17) 294 (77) 151 (40) 76 (20) 155 (41) 132 (35) 194 (51) 56 (15) 61 (16) 68 (18) 253 (66) 124 (32) 108 (28) 150 (39) 132 (54) 106 (43) 7 (3) |
(a) High perceived risk corresponds to ratings >5
(b) n=349. Excludes participants who did not answer or answered ambiguously
(c) n=343. Excludes participants who did not answer or answered ambiguously
(d) n=244. Excludes participants who answered “I have no children”
(e) Full question is as follows: If your child had a fever after travelling to Africa, Latin America, or Asia and you took him/her to see the doctor; do you agree with the following statement.
